# Rapid On-Site Detection Method for White Spot Syndrome Virus Using Recombinase Polymerase Amplification Combined With Lateral Flow Test Strip Technology

**DOI:** 10.3389/fcimb.2022.889775

**Published:** 2022-07-15

**Authors:** Tianmeng Zhang, Xia Liu, Xiaohan Yang, Feixue Liu, Haitao Yang, Xueqing Li, Huimiao Feng, Xinyu Wu, Ge Jiang, Hui Shen, Jingquan Dong

**Affiliations:** ^1^ Jiangsu Key Laboratory of Marine Bioresources and Environment, Co-Innovation Center of Jiangsu Marine Bio-Industry Technology, Jiangsu Key Laboratory of Marine Pharmaceutical Compound Screening, Jiangsu Ocean University, Lianyungang, China; ^2^ Department of Laboratory Medicine, The Second People’s Hospital of Lianyungang City, Lianyungang, China; ^3^ College of Life Science and Technology, Huazhong University of Science and Technology, Wuhan, China; ^4^ Marine Fisheries Research Institute of Jiangsu, Nantong, China

**Keywords:** white spot syndrome virus, recombinase polymerase amplification, lateral flow strip, rapid on-site detection, shrimp

## Abstract

The white spot syndrome virus is the most destructive virus threatening the shrimp industry worldwide, causing hundreds of millions of dollars in economic losses each year. There is currently no specific medicine to treat it. Therefore, rapid and accurate detection of WSSV is of great significance for controlling its spread and reducing economic losses. Traditional detection methods, such as polymerase chain reaction (PCR) and quantitative fluorescent PCR, rely on laboratory equipment and are not suitable for field testing. In this study, recombinase polymerase amplification (RPA) combined with a lateral flow strip (LFS) was developed. This method targets the entire genome and designs primers and probes accordingly. The detection can be completed in 30 min at 37°C, and the detection limit of each reaction is 20 copies, which is much more sensitive than other detection methods. The RPA-LFS method is highly specific to the white spot syndrome virus and has no cross-reactivity with other common shrimp viruses or pathogens. In total, 100 field samples were tested and compared to the real-time PCR method. Both methods detected 8 positive results, and the positive detection rate was 100%. The method was fast, simple, specific, and sensitive. It does not rely on laboratory equipment and has broad application prospects for in-field detection, especially in remote areas with underdeveloped medical equipment.

## Introduction

Shrimp is the largest aquaculture species in the world. The world’s shrimp exports exceeded 20 billion US dollars in 2015, and by 2017, the annual output exceeded 5.5 million tons (Asche et al., 2020), but various sudden infections pose a serious threat to the global shrimp industry (Flegel, 2019). White spot disease (WSD) is the most serious infectious disease of prawns worldwide (Li and Wang, 2021). It is caused by the white spot syndrome virus (WSSV), which is an enveloped double-stranded linear DNA virus of the family Nimaviridae with a genome of approximately 300 kilobase pairs (kbp) (van Hulten et al., 2001; Leu et al., 2009). In addition, it can infect other aquatic crustaceans such as crabs, crayfish, etc. (de Souza Valente et al., 2020; Chen et al., 2021; Yang et al., 2022). The world’s first case of WSSV was reported in Taiwan in 1992 and spread rapidly in Southeast Asia (Zhu et al., 2019), and in 1994, WSSV spread to most shrimp farms in Asia. By 1995, WSSV had spread to the eastern United States *via* freezing commodities, and by 1999, it had spread to Pacific nations (Lightner et al., 2012). The World Organization for Animal Health classifies it as a destructive crustacean virus. WSSV-infected prawns have decreased vitality and hollow gastrointestinal tracts, and the shells are easily peeled off, with obvious white spots that can be seen on the shells. WSSV is extremely harmful to shrimp, and the mortality rate can reach 100% within 3 to 10 days after clinical symptoms appear. The world loses more than 3 billion US dollars from the disease every year, and this loss is increasing (Millard et al., 2020). Unfortunately, there is no effective method to treat WSD. Therefore, strict biosecurity measures, corresponding detection methods, and extinction measures are particularly important for the rapid and accurate identification of WSSV to prevent the disease and protect the growth environment of shrimps.

At present, there are many detection methods for WSSV. The international standard is the detection method of nest PCR recommended by OIE (Park et al., 2013). The traditional quantitative analysis of WSSV primary cell culture is relatively cumbersome. Laboratory requirements, particularly aseptic environment requirements, are extremely strict. The entire culture process takes 2–3 weeks, and the results after infection require transmission electron microscope observation. The detection cycle is long and time-consuming (Tapay et al., 1997). The emergence of molecular diagnostic technologies, such as PCR technology and qPCR, has solved the problem of excessive time consumption. These technologies design primers for specific genes, and after strict testing, the results show high specificity and sensitivity (Kim et al., 1998; Mendoza-Cano and Sánchez-Paz, 2013). However, reading results at higher temperatures and relayed on PCR instruments or fluorescence quantitative equipment require well-trained professionals. The emergence of isothermal technology has solved the temperature dependence problem, such as loop-mediated isothermal amplification (LAMP) technology and RPA (Xia et al., 2014; Waiwijit et al., 2015). However, LAMP detection requires stricter primers and is prone to false-positive results. These shortcomings make them difficult to use in the field of conventional surveillance (Schneider et al., 2019; Trung et al., 2022) . Now, some new detection methods have emerged, such as the rapid detection of WSSV using lateral flow technology combined with phage-displayed peptides as biorecognition probes (Kulabhusan et al., 2017) and the electrochemical detection of WSSV using silicone rubber disposable electrodes embedded with graphene quantum dots and gold nanoparticles (Takemura et al., 2020). In addition, CRISPR systems have also been implicated in the detection of WSSV. However, although these methods have improved sensitivity when combined with emerging technologies, the complex technology limits them to rapid field detection (Chaijarasphong et al., 2019). Therefore, to avoid the spread of the epidemic caused by the long detection time, shrimp farming must reduce detection costs and workload, facilitate real-time monitoring of the shrimp farm to cut off the pathogen in time, prevent economic losses, and establish a rapid on-site inspection method. Furthermore, this method is particularly suitable for ports or remote areas with underdeveloped medical equipment.

**Figure 1:**
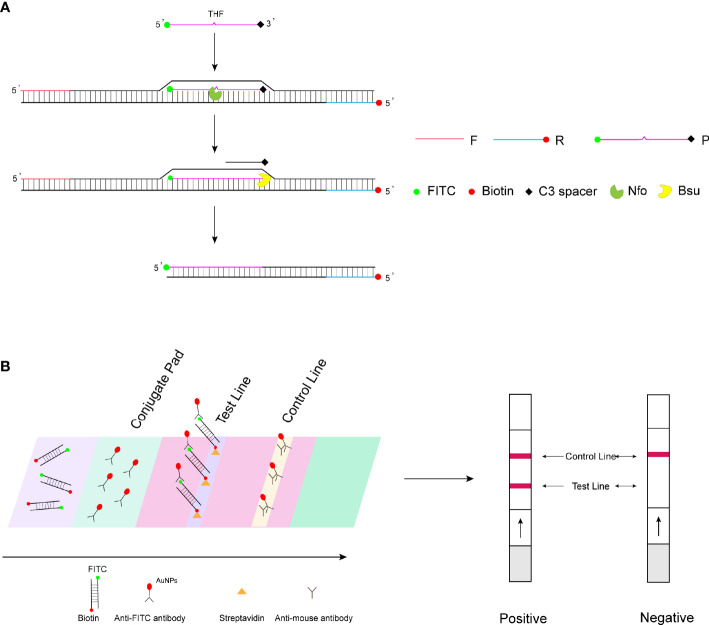
RPA-LFS detection mechanism diagram. **(A)** Principles of RPA amplification. **(B)** Visualization of lateral flow test strips.

Recombinase polymerase amplification (RPA) is an isothermal nucleic acid amplification technology reported in 2006 (Piepenburg et al., 2006). The principle of RPA amplification relies on the formation of a complex of recombinase and primers to find homologous sequences in double-stranded DNA, followed by a strand exchange reaction and exponential amplification by the polymerase. The RPA amplification products can be visualized by agarose gel electrophoresis, real-time fluorescence monitoring, and lateral flow strip assay. The study wanted to achieve rapid on-set detection of WSSV by combining RPA amplification with lateral flow test strips. Therefore, in this study, the 5′ end of the probe was labeled with a fluorescent group (fluorescein isothiocyanate (FITC)) and the 5′ end of the reverse primer was labeled with biotin, with the aim of amplifying a target product with both a fluorescent group (FITC) and biotin. For the LFS assay, the control line on the strip is labeled with the anti-mouse antibody and the test line with streptavidin. The FITC end of the double-labeled RPA amplification product binds to the AuNPs of the bonding pad (wrapped by the anti-FITC antibody), and then the biotin end binds to the streptavidin on the test line, showing a red positive band, while the AuNPs not bound to the amplification product bind to the anti-mouse antibody on the control line, showing a red color (**Figure 1**). Since no temperature control equipment is required, RPA-LFS can truly realize portable and rapid on-site pathogen detection. This method has been widely proven to be a fast and accurate method for the rapid detection of pathogens in the field. Fan et al. (2020) applied RPA-LFS to African swine fever virus detection; Xiong et al. (2020) applied CRISPR-Cas12-RPA to coronavirus disease (COVID-19) detection; and Li et al. (2019) applied RPA-LFS to rapid field detection of *Salmonella* in food.

In this study, RPA-LFS was established to detect WSSV with high specificity and sensitivity. The primers and probes were designed to specifically recognize WSSV only but not other viruses or pathogens. The RPA-LFS method can be performed within 30 min at a constant temperature of 37°C. Compared with other methods, this method is fast, simple, specific, and sensitive and is propitious to rapid on-site detection of WSSV.

## Materials and Method

### Strain Collection and DNA Extraction

The shrimp infected naturally with WSSV was obtained from the Jiangsu Institute of Oceanology and Marine Fisheries (Nantong, China). *Vibrio parahaemolyticus*, *Vibrio cholerae*, *Vibrio alginolyticus*, *Vibrio harveyi*, *Vibrio cholerae*, *Acute hepatopancreatic necrosis disease* (AHPND), *Enterocytozoon hepatopenaei* (EHP), *Vibrio ichthyoenteri*, *Vibrio rotiferianus*, *Vibrio mimicus*, *Vibrio shilonii*, *Vibrio splendidus*, *Pseudomonas aeruginosa*, *Bacillus cereus*, *Salmonella typhimurium*, and *Listeria monocytogenes* were preserved in the laboratory and confirmed by sequencing. The DNA extraction kit was used for DNA isolation and purification (Tiangen Biotech Co., Ltd., Beijing, China).

### Construction of Recombinant Plasmid Standard

The DNA of shrimp infected with WSSV was used as the template, and the target gene VP28 was amplified by PCR to obtain the target fragment of 140 bp (F-AGGTGTGGAACAACAC ATCAAG, R-TGCCAACTTCATCCTCATCA) (Xia et al., 2014), which was cloned into the pMD18-T vector (Takara Biomedical Technology Co. Ltd., Beijing, China). The recombinant vector was transferred into competent *Escherichia coli* DH5α cells and extracted with a plasmid extraction kit (Tiangen). The recombinant plasmid was verified by sequencing. Quantification was performed using a Qubit 4 fluorometer, and the copy number was calculated based on the concentration and base number of the recombinant plasmid. The standard plasmid was diluted by the 10-fold gradient. The standard curve was established according to the correlation between Ct value and copy number, and then the quantity of WSSV standard plasmids was calculated.

### Design of RPA Primers

The whole-genome sequence of WSSV (GenBank No. NC_003225.3) was entered into NCBI Primer-BLAST (https://www.ncbi.nlm.nih.gov/tools/primer-blast). According to the principle of RPA, RPA requires a longer primer length (approximately 30–35 nucleotides) compared to conventional PCR primers. The primer design should consider a variety of factors, including hairpin structure, mismatch, primer–dimer, and primer efficiency. Therefore, the following criteria were considered for the implementation of Primer-BLAST in this study. The product size was set between 100 and 500 bases. Primer size was set at 30 to 35 bases. The primer GC content was at a minimum of 20 and maximum of 70. The maximum self-complementarity was set at 1. Other parameters were set by default. The 5′ end of the reverse primer was modified with biotin. Primers were synthesized by General Biosystems Co. Ltd., Anhui, China.

### RPA Program and Electrophoresis

The RPA reaction was performed using the TwistAmp^®^ Liquid DNA Amplification Kit (TwistDx Inc., Maidenhead, UK). The 50-μl reaction system consists of 25 μl of 2× reaction buffer, 5 μl of 10× basic mix, 2.5 μl of 20× core mix, 2.1 μl of each primer (10 μM), 1 μl of template, and 9.8 μl of distilled water. Finally, 2.5 μl of magnesium acetate (280 mM) was used to initiate the reaction. The mixture was reacted at 37°C for 30 min. RPA reaction results were read using 1.5% agarose gel electrophoresis.

### Design of Probes

The specificity and sensitivity of RPA reactions using this probe can be improved. Based on primers with good specificity, probe design was performed between the two primers using Primer Premier 5 software. The size of the probe was set to a minimum of 46 and a maximum of 52. The lowest GC content was 20, and the highest was 80. The maximum score for hairpins is 9. The primary dimer fraction is set to a maximum value of 9. The maximum Poly-X setting was 5. Other parameters are defaulted to follow the system. The 3′ end of the probe was modified with a C3 spacer to prevent strand growth. There is a tetrahydrofuran (THF) site on the 31st base of the probe to facilitate cleavage by the nfo enzyme. The 5′ end of the probe was modified with FITC. The probe was synthesized by Anhui General Biosystems Co., Ltd., China.

### RPA-LFS Procedure

The RPA reaction was performed according to the TwistAmp DNA amplification nfo kit (TwistDx Inc., Maidenhead, UK). To form a 50-μl reaction system, 2.1 μl of each primer (10 μM), 0.6 μl of probe (10 μM), 1 μl of template, and other standard reaction components were mixed. Furthermore, 2.5 μl of magnesium acetate (280 mM) was added to initiate the reaction. The reaction mixture was incubated for 40 min under 37°C. Five microliters of the amplification product was used for the LFS visualization readout (Ustar Biotechnologies Ltd., Hangzhou, China).

### Quantitative PCR

A pair of specific primers targeting the VP28 gene (Xia et al., 2014) of WSSV was used for qPCR analysis. To make up a 20-μl reaction system, 0.4 μl of primer (10 μM), 1 μl of template, 10 μl of Monamp TM SYBR Green qPCR mix, and 8.2 μl of nuclease-free water were mixed. The cycle program was 95°C for 5 min, followed by 35 cycles of 94°C for 30 s, 61°C for 30 s, 72°C for 30 s, and an extension of 5 min at 72°C on Rochel LightCycler 480 II qPCR machine. The melting curve analysis was set as the default.

## Results

### Standard Curve for Copy Number Determination

**Figure 2:**
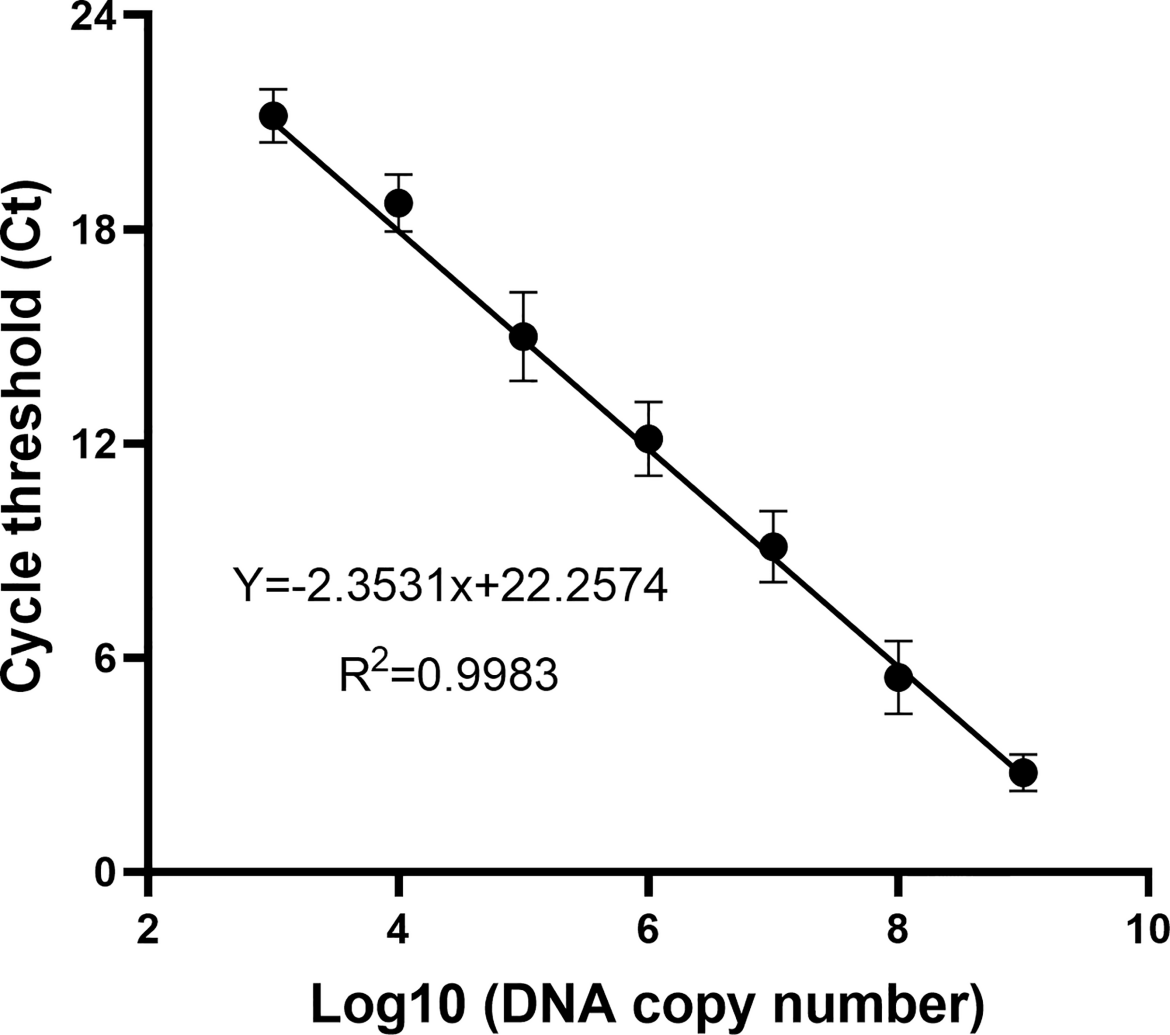
The standard curve of the recombinant plasmid was obtained by a qPCR assay. Of the recombinant plasmid, 2 × 10^9^–10^3^ copies/ml were used as the template for the reaction. A standard curve of the correlation between DNA copy number and qPCR cycle threshold (Ct) value was constructed using GraphPad Prism 8.0. *R* ^2^ represents the degree of fit of the regression line.

To determine the DNA copy number of WSSV, the recombinant plasmid containing the VP28 gene was diluted from 2 × 10^9^ to 2 × 10^3^ copies/ml according to a 10-fold gradient and used as a template for qPCR amplification. The result is shown in **Figure 2**. The DNA copy number and Ct value showed a good correlation (*R* ^2^ = 0.9983), indicating that the copy number of WSSV was determined.

### Preliminary Primer Screening of WSSV Detection

**Figure 3:**
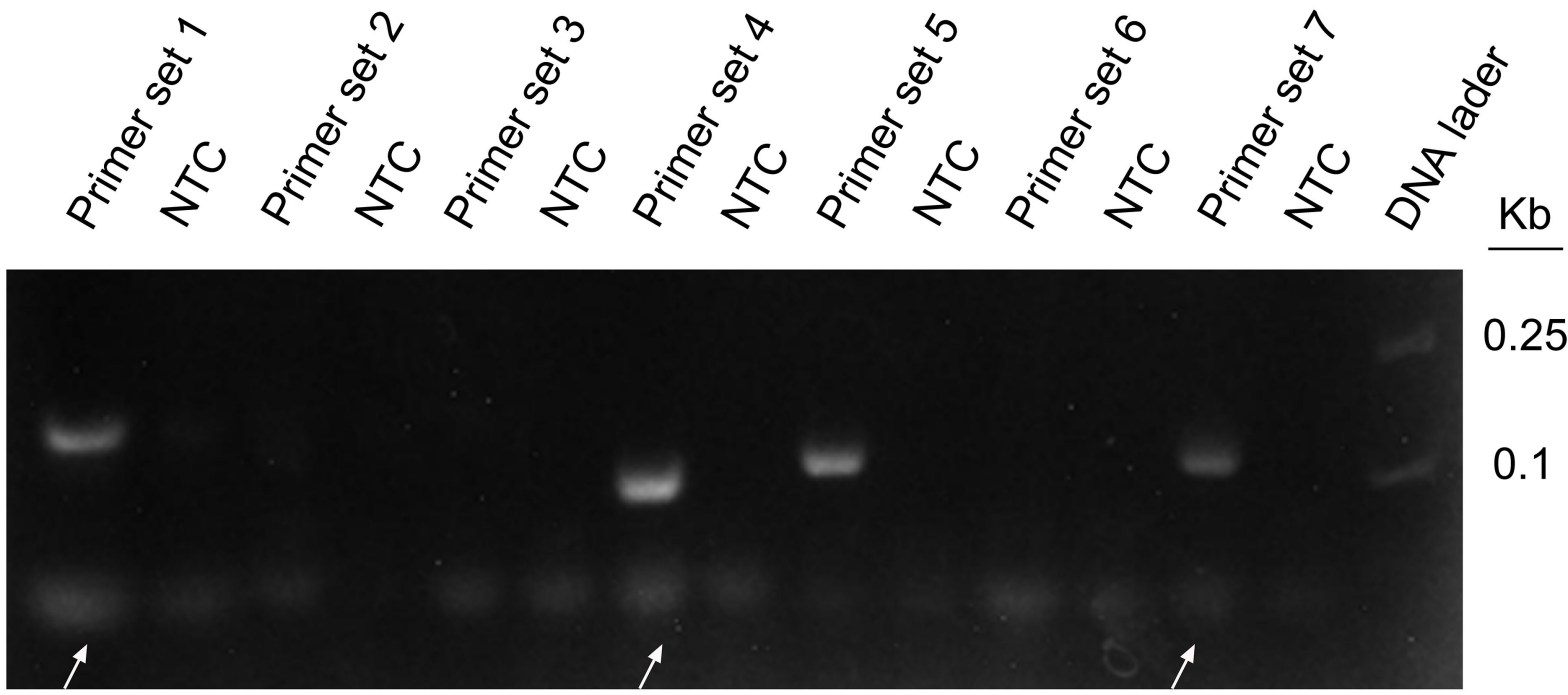
RPA amplification combined with agarose gel electrophoresis for preliminary primer screening; 1 µl of 2 × 10^9^ copies/ml was used as the template for the reaction. Lanes 1, 3, 5, 7, 9, 11, and 13 represent 7 different primer combinations. Lane NTC represents the negative control without template DNA in the left lane. White arrows represent the primer dimers. The primer names are shown at the top of each band.

Seven pairs of primers were designed for the genome of WSSV. The purpose of using WSSV as the template for RPA amplification is to screen primer pairs with good amplification performance. The agarose gel electrophoresis results showed that primer sets 1, 4, 5, and 6 had obvious specific amplified fragments, while the other three pairs did not have amplified fragments, and primer sets 1 and 7 amplified bands were not obvious (**Figure 3**). Primer set 4 had obvious primer dimers, so primer set 5 was selected for subsequent experiments. All primers are shown in **Table 1**.

**Table 1:** Primers and probes for this study.

Name	Sequence (5′-3′)	Length (bp)	Region
F1	TACTACTGAAATCTTCTAAACACACTGGAC	157	32618–32774
R1	TTGGGGTATATTGCATCCTCAAATTATCCT		
F2	TTACTGTTTTTCTGTTCATCCCTCTTTCCT	133	98304–98436
R2	GATCTATCCAACTGTGTTTTGTAATGGGTG		
F3	GTCGGGTAAATAGATCCTTGTTAGATTTTT	187	141874–142060
R3	TCAGACCCTTTAGTCAACACATTATTTCTATC		
F4	TAAATATACTGCTCTGTCGAAATTGTTCGTTATTG	115	191757–191871
R4	AAAATATGTCCATGTATCTAAAGGGTTTGCTG		
F5	CAAAAGAAAATTCAAACTCCTCAACTCATT	137	239621–239757
R5	ACATTATTTCACTCATAAGAACACTCCTTC		
F6	CAAATCTTCTGGTAGTCTTTTGAGCCAATC	102	284132–284233
R6	ACGTCATTATTTCTTCAACATCATTCACAA		
F7	TGTTCAAATCTTACTACATAACCCAAGAAA	129	308308–308436
R7	CATATACAACATGACTAACCAACCAAAAGAAA		
P	CAAAAGAAAATTCAAACTCCTCAACTCATT[THF]TTGGTTCATAACAACA	46	239621–239667
Pm	CAATAGACAATTCAAACTCCTCAACTCATT[THF]TTGGTTCATAACAACA	46	239727–239757
R5m	ACATTACTTCACTCATAAGATCACTCCTTC	30	239621–239757
F5-1	AGTTTCTACCATTGGAGACTATGTCTTATCA	31	239462–239493
F5-2	TACCATTGGAGACTATGTCTTATCAAACCCC	31	239468–239499
F5-3	AGAGTATAGTTTCTACCATTGGAGACTATGTC	32	239455–239487
F5-4	TTGTGACTCTGAGACGTACACCAAACCTATA	31	239408–239439
F5-5	GTGACTCTGAGACGTACACCAAACCTATACCG	32	239410–239442
F5-4/Pm/R5m	/	350	239408–239757

### Probe Screening

**Figure 4:**
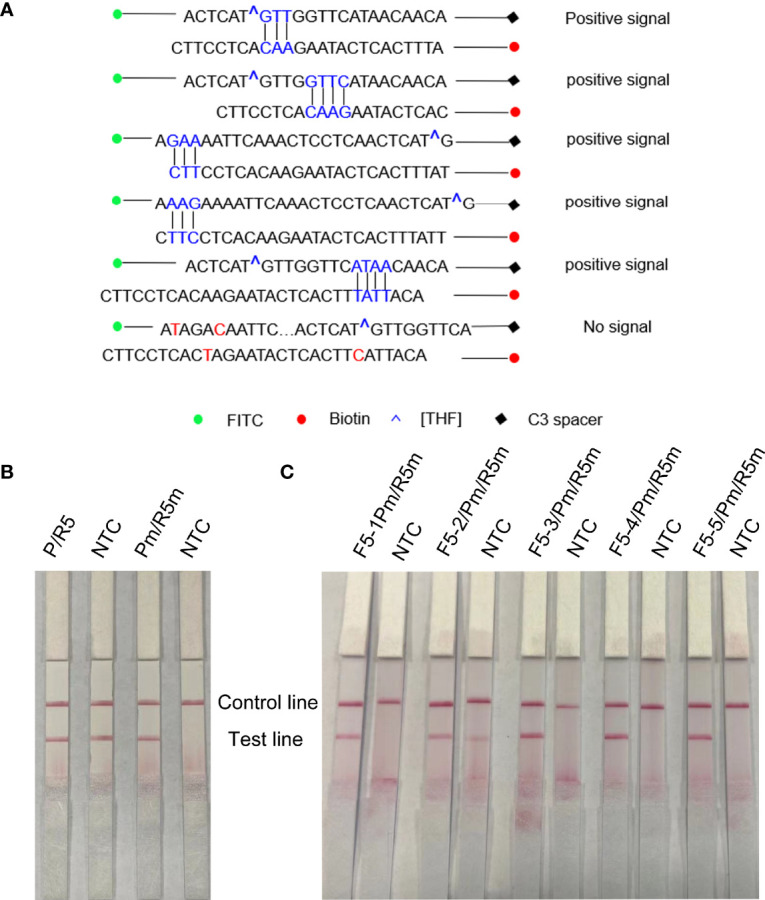
Screening of forwarding primers using RPA-LFS; 1 µl of 2 × 10^9^ copies/ml was used as the template for the reaction. **(A)** Sequence analysis of primer–probe combination (P/R5) and its modification; the number of consecutive matching bases is greater than three and is connected by vertical lines. **(B)** RPA-LFS reaction was performed using reverse primers and probes. **(C)** Five forward primers were combined with probe and reverse primer for WSSV amplification, respectively. NTC was a no-template negative control. The primer names are shown at the top of each band.

As F5 and R5 primers have been proven to have good specificity, the forward primer F5 was extended from 5′ to 3′ to 17′bp as a probe (including THF site) in this study, and the probe (P) and reverse primer (R5) were used for amplification to observe whether there was a specific amplification in the negative control. The RPA-LFS results showed that the negative control was amplified in **Figure 4B**, that is, false positives existed. In order to solve this problem, the secondary structure between primers was analyzed. First, cross-dimer analysis between the probe and the reverse primer was performed using Primer 5.0 software. It shows 5 places of consecutive base matches between the probe and reverse primer (**Figure 4A**). The RPA-LFS detection of the reverse primer and probe showed that amplification occurred in the negative control (**Figure 4B**, left), Therefore, at the continuous base, the probe and the reverse primer mismatch two bases, respectively. Cross-dimmers were fully eliminated in theory (**Figure 4A**). The introduction of mismatched bases did reduce and eliminate false-positive results in the RPA-LFS method (**Figure 4B**, right), thus demonstrating that the R5m/Pm combination can be used for subsequent experiments. The information on modified primers and probes are shown in **Table 1**.

### Forward Primer Screening

Five forward primers (F5-1, F5-2, F5-3, F5-4, and F5-5) were designed according to the reverse primers and probes, which were combined with R5m and Pm, respectively, and detected by RPA-LFS. The results are shown in **Figure 4C**. Using WSSV as the template, all 5 pairs were positive for amplification, but F5-2/R5m/Pm was a false positive. Among the other 4 pairs of primers, the band containing F5-4/R5m/Pm was the darkest, that is, the F5-4/R5m/Pm combination is the best primer–probe combination, and the combination was applied to other subsequent experiments.

### Optimization of Reaction Conditions

**Figure 5:**
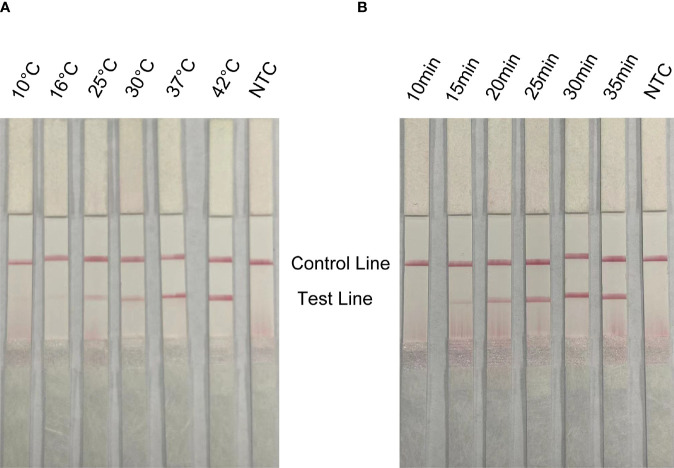
Optimization of reaction conditions for RPA-LFS; 1 µl of 2 × 10^9^ copies/ml was used as the template for the reaction. **(A)** The RPA-LFS test was performed at different reaction temperatures ranging from 10°C to 42°C. **(B)** The RPA-LFS tests were carried out at different reaction times ranging from 10 to 35 min. NTC was a template-free negative control. The time and temperature of the reaction are shown at the top of the strip, respectively.

In order to apply the RPA-LFS to rapid field detection, the reaction temperature and reaction time of RPA reaction conditions were optimized. The white spot syndrome virus was used as a template for RPA amplification at 10°C to 42°C (**Figure 5A**). The brightness of the reaction strip increases with the increase of the reaction temperature. The darker red stripe begins to appear at 37°C, and the deepest red stripe appears at 42°C. Considering the need for rapid on-site detection, 37°C is determined as the best reaction temperature. In addition, the optimal reaction time was selected within 10 to 35 min (**Figure 5B**). At 15 min, the red band of the test line appears, and the deepest band appears at 30 min. Therefore, the best reaction temperature measured by RPA-LFS was 37°C and the best reaction time was 30 min.

### The Specificity of Detection of the RPA-LFS Method

**Figure 6:**
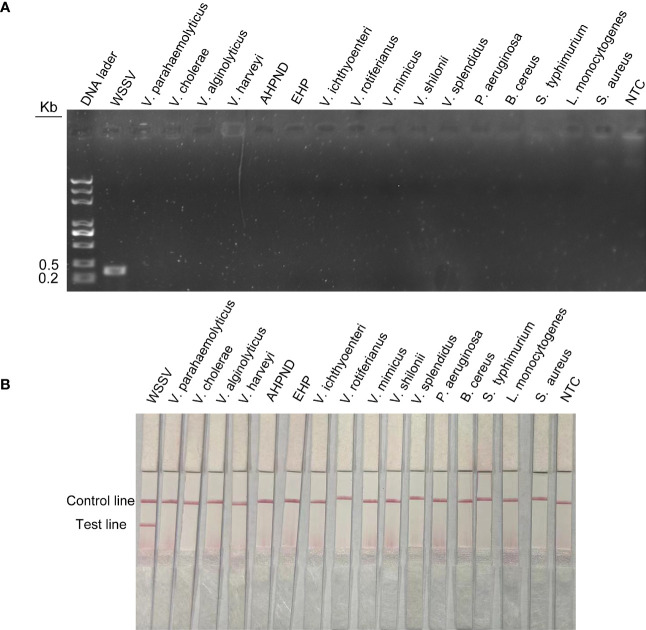
Specific detection of RPA-LFS reaction. **(A)** Result of RPA-amplified agarose gel electrophoresis. **(B)** Result of RPA-amplified LFS. Of the standard plasmid, 1 µl of 2 × 10^9^ copies/ml was used as the template and reacted for 30 min at 37°C. The remaining templates are the common shrimp disease pathogens and the pathogens widely distributed in nature, which are *Vibrio parahaemolyticus*, *Vibrio cholerae*, *Vibrio alginolyticus*, *Vibrio harveyi*, AHPND, EHP, *Vibrio ichthyoenteri*, *Vibrio rotiferianus*, *Vibrio mimicus*, *Vibrio shilonii*, *Vibrio splendidus*, *Pseudomonas aeruginosa*, *Bacillus cereus*, *Salmonella typhimurium*, *Listeria monocytogenes*, and *Staphylococcus aureus*. NTC was a template-free negative control. The names of the bacterial species are shown at the top of each band.

To assess the specificity of the method for WSSV, viruses or microorganisms associated with shrimp diseases, such as WSSV, EHP and AHPND, were tested. In addition, common pathogenic bacteria in the aquatic environment, such as *Vibrio parahaemolyticus*, *Vibrio haemolyticus*, *Vibrio cholerae*, *Pseudomonas aeruginosa*, *Bacillus cereus* and *Listeria monocytogenes*, were tested. The results of agarose gel electrophoresis showed that the above primer pairs were very specific and could accurately exclude all curing bacteria except WSSV (**Figure 6A**). The results of LFS were consistent with agarose gel electrophoresis. The primer–probe combination used in this study could only amplify WSSV, and no bands of other pathogens were found in the test lines (**Figure 6B**). Therefore, RPA-LFS is suitable for WSSV detection with good specificity and no false-positive results.

### Limit of Detection of the RPA-LFS Method

**Figure 7:**
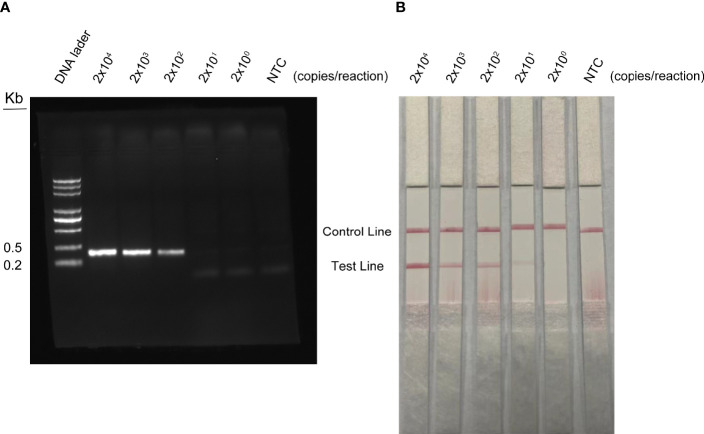
Detection limit of RPA-LFS reaction. One microliter of WSSV (2 × 10^7^ to 2 × 10^3^ copies/ml) was used as a template and reacted at 37°C for 30 min. **(A)** The result of the agarose gel electrophoresis. **(B)** The result of the LFS analysis. The amount of WSSV is shown at the top of each strip. NTC was a template-free negative control.

The detection limit of the RPA method was conducted with different concentrations of WSSV. DNA was extracted from shrimp infected with WSSV and quantified by qPCR using standard curves. The quantified DNA was diluted with a 10-fold gradient from 2 × 10^7^ to 2 × 10^3^ copies/ml, and 1 µl of WSSV was taken for RPA amplification. The agarose gel electrophoresis of RPA showed that the detection limit of WSSV was 2 × 10^2^ copies/reaction (**Figure 7A**). The results of RPA-LFS showed that red bands were visible on the test line at a concentration of 2 × 10^4^ to 2 × 10^0^ copies/reaction (**Figure 7B**). As the number of copies decreased, the red brightness of the test line gradually decreased, with a weak red band appearing at 2 × 10^1^ copies/reaction. Therefore, the results of this study show that the LFS method is more sensitive than agarose gel electrophoresis, and the detection limit of the RPA-LFS was 20 copies/reaction.

### Clinical Sample Detection

In this study, 80 shrimp samples of different types and 20 aquaculture water samples from different farms in different regions were collected for clinical sample testing. The surfaces of the shrimp samples were first disinfected with ethanol, and then the tissues were cut into small pieces and ground into a homogenate using an electric grinder and a boiling water bath. Finally, 1 µl was used as the assay template for RPA-LFS and qPCR reactions. The results are shown in **Table 2**. Both methods could detect 8 infected shrimp samples, and the consistency rate of WSSV detection was 100%. However, neither method detected WSSV in 20 water samples.

**Table 2:** Detection of WSSV in clinical samples.

Sample number	RPA-LFS assay	qPCR assay		Sample number	RPA-LFS assay	qPCR assay	
		Result	Ct (*n* = 3)			Result	Ct (*n* = 3)
No. 1	−	−	−	No. 41	−	−	−
No. 2	−	−	−	No. 42	−	−	−
No. 3	−	−	−	No. 43	−	−	−
No. 4	−	−	−	No. 44	−	−	−
No. 5	−	−	−	No. 45	−	−	−
No. 6	+	+	12.84	No. 46	**+**	**+**	13.73
No. 7	−	−	−	No. 47	−	−	−
No. 8	−	−	−	No. 48	−	−	−
No. 9	−	−	−	No. 49	−	−	−
No. 10	−	−	−	No. 50	−	−	−
No. 11	−	−	−	No. 51	**+**	**+**	32.63
No. 12	−	−	−	No. 52	−	−	−
No. 13	**+**	**+**	32.51	No. 53	−	−	−
No. 14	−	−	−	No. 54	−	−	−
No. 15	−	−	−	No. 55	−	−	−
No. 16	−	−	−	No. 56	−	−	−
No. 17	−	−	−	No. 57	−	−	−
No. 18	−	−	−	No. 58	**+**	**+**	33.83
No. 19	**+**	**+**	16.00	No. 59	−	−	−
No. 20	−	−	−	No. 60	−	−	−
No. 21	−	−	−	No. 61	−	−	−
No. 22	**+**	**+**	31.65	No. 62	−	−	−
No. 23	−	−	−	No. 63	−	−	−
No. 24	−	−	−	No. 64	−	−	−
No. 25	−	−	−	No. 65	−	−	−
No. 26	−	−	−	No. 66	−	−	−
No. 27	−	−	−	No. 67	−	−	−
No. 28	−	−	−	No. 68	−	−	−
No. 29	−	−	−	No. 69	−	−	−
No. 30	−	−	−	No. 70	−	−	−
No. 31	−	−	−	No. 71	−	−	−
No. 32	−	−	−	No. 72	−	−	−
No. 33	−	−	−	No. 73	−	−	−
No. 34	−	−	−	No. 74	−	−	−
No. 35	−	−	−	No. 75	−	−	−
No. 36	−	−	−	No. 76	**+**	**+**	13.66
No. 37	−	−	−	No. 77	−	−	−
No. 38	−	−	−	No. 78	−	−	−
No. 39	−	−	−	No. 79	−	−	−
No. 40	−	−	−	No. 80	−	−	−

“+” positive result; “−” negative result.

## Discussion

WSSV is an important pathogen endangering aquaculture worldwide. It killed a large number of crustaceans and shrimp. In particular, it poses a huge threat to prawn farming. WSSV spreads in various ways, with fast spread and high mortality. With no effective treatments on the market, it causes very serious economic losses worldwide every year. Therefore, fast and accurate on-site detection methods are of great significance for the early diagnosis of WSSV infection, real-time monitoring, and timely cut off of pathogens in prawns farming and remote epidemic areas with underdeveloped medical detection equipment.

In recent years, many PCR-based molecular diagnostic techniques have emerged for the detection of WSSV. For example, qPCR technology can detect up to 12 copies per sample of WSSV (Mendoza-Cano and Sánchez-Paz, 2013), and the detection limit of insulated isothermal PCR (iiPCR) is 17 copies/reaction (Tsai et al., 2014); the detection limit of real-time quantitative LAMP is 100 copies of template DNA (Mekata et al., 2009); real-time RPA can detect WSSV in 5 copies/μl (Xia et al., 2014); and the CRISPR-based SHERLOCK method realizes the single-copy detection of WSSV (Sullivan et al., 2019). In this study, the standard plasmid of WSSV genomic DNA was diluted from 2 × 10^4^ to 2 × 10^0^ copies/ml, and the RPA-LFS sensitivity was 20 copies/reaction. Although the RPA-LFS technology used in this study is not as sensitive as SHERLOCK and real-time RPA technology, it avoids the high-cost detection of SHERLOCK technology and the real-time RPA detection of equipment dependence. Therefore, RPA-LFS is more suitable for the rapid field detection of WSSV (Mustafa and Makhawi, 2021).

In this study, primer–probe groups were determined through a rigorous selection and screening process. We did not use the traditional design of probes based on the amplification range of forward and reverse primers, but first designed reverse primers and probes. Since the reverse primer and probe are modified with biotin and fluorophore respectively in the RPA-LFS reaction, the double-labeled products amplified by the reverse primer and probe can be colored on the band. If the specificity and conservation of this critical region are selected well, the specificity and sensitivity of the final results will be good. Therefore, after confirming the reverse primers and probes, the forward primers can be designed. This method not only ensures the specificity and sensitivity of the RPA-LFS reaction but also saves a lot of time for primer design.

For the specific detection of the RPA-LFS method, this study used common shrimp diseases as detection templates, including EHP, AHPND, *Vibrio alginolyticus*, *Vibrio parahaemolyticus*, *Vibrio cholerae*, and other common aquatic pathogens. *Pseudomonas aeruginosa*, *Bacillus cereus*, and *Salmonella typhimurium* are widely distributed in nature. The selection of pathogens from different sources can better ensure the specificity of the RPA-LFS method for the detection of WSSV and the RPA-LFS method for the detection of clinical samples. The RPA-LFS detection technology can detect 8 positives in 100 clinical samples, and the consistency rate with the qPCR detection results is 100%. Therefore, this study believes that the WSSV detection method provided by RPA-LFS technology has high specificity and sensitivity and can be applied to rapid detection in the field.

## Conclusions

This research established the RPA-LFS method to detect WSSV in shrimp. This method is fast, simple, specific, and sensitive. It can be completed within 30 min at room temperature. It is suitable for grassroots breeding units and first-line ports. The on-site inspection is also of great significance for remote areas where medical equipment is scarce.

## Data Availability Statement

The raw data supporting the conclusions of this article will be made available by the authors, without undue reservation.

## Author Contributions

JD, HS and GJ designed the experiment. TZ, XL and XY performed the experiment, FL, HY and XQL performed the data analysis, HF and XW were responsible for the software application and data organization, and TZ wrote the manuscript". All authors contributed to the article and approved the submitted version.

## Funding

The authors thank the Basic Science (Natural Science) Research Project of Higher Education of Jiangsu Province (No. 21KJB230001), the Open-end Funds of Jiangsu Key Laboratory of Marine Pharmaceutical Compound Screening (No. HY202101), Research project on high-quality development of fishery in Yancheng, China(YCSCYJ2021005); Nantong Science and Technology Project (JS2021089, MS12020059) and the Priority Academic Program Development of Jiangsu Higher Education Institutions of China for financial support.

## Conflict of Interest

The authors declare that the research was conducted in the absence of any commercial or financial relationships that could be construed as a potential conflict of interest.

## Publisher’s Note

All claims expressed in this article are solely those of the authors and do not necessarily represent those of their affiliated organizations, or those of the publisher, the editors and the reviewers. Any product that may be evaluated in this article, or claim that may be made by its manufacturer, is not guaranteed or endorsed by the publisher.

## References

[B1] Asche F. Anderson J. L. Botta R. Kumar G. Abrahamsen E. B. Nguyen L. T.. (2020). The Economics of Shrimp Disease. J. Invertebr. Pathol. 186, 107397. doi: 10.1016/j.jip.2020.107397 32446865

[B2] Chaijarasphong T. Thammachai T. Itsathitphaisarn O. Sritunyalucksana K. Suebsing R. (2019). Potential Application of CRISPR-Cas12a Fluorescence Assay Coupled With Rapid Nucleic Acid Amplification for Detection of White Spot Syndrome Virus in Shrimp. Aquaculture 512. doi: 10.1016/j.aquaculture.2019.734340

[B3] Chen C. Shen J. L. Wang T. Yang B. Liang C. S. Jiang H. F.. (2021). Ophiopogon Japonicus Inhibits White Spot Syndrome Virus Proliferation *In Vivo* and Enhances Immune Response in Chinese Mitten Crab Eriocheir Sinensis. Fish Shellfish Immunol. 119, 432–441. doi: 10.1016/j.fsi.2021.10.028 34688864

[B4] de Souza Valente C. Rodiles A. Freire Marques M. R. Merrifield D. L. (2020). White Spot Syndrome Virus (WSSV) Disturbs the Intestinal Microbiota of Shrimp (Penaeus Vannamei) Reared in Biofloc and Clear Seawater. Appl. Microbiol. Biotechnol. 104 (18), 8007–8023. doi: 10.1007/s00253-020-10816-4 32789745

[B5] Fan X. Li L. Zhao Y. Liu Y. Liu C. Wang Q.. (2020). Clinical Validation of Two Recombinase-Based Isothermal Amplification Assays (RPA/RAA) for the Rapid Detection of African Swine Fever Virus. Front. Microbiol. 11, 1696. doi: 10.3389/fmicb.2020.01696 32793160 PMC7385304

[B6] Flegel T. W. (2019). A Future Vision for Disease Control in Shrimp Aquaculture. J. World Aquacult. Soc. 50 (2), 249–266. doi: 10.1111/jwas.12589

[B7] Kim Kim Sohn Sim Park Heo. (1998). Development of a Polymerase Chain Reaction (PCR) Procedure for the Detection of Baculovirus Associated With White Spot Syndrome (WSBV) in Penaeid Shrimp. J. Fish Dis. 21 (1), 11–17. doi: 10.1046/j.1365-2761.1998.00324.x 29739168

[B8] Kulabhusan P. K. Rajwade J. M. Sahul Hameed A. S. Paknikar K. M. (2017). Lateral Flow Assay for Rapid Detection of White Spot Syndrome Virus (WSSV) Using a Phage-Displayed Peptide as Bio-Recognition Probe. Appl. Microbiol. Biotechnol. 101 (11), 4459–4469. doi: 10.1007/s00253-017-8232-6 28349164

[B9] Leu J. H. Yang F. Zhang X. Xu X. Kou G. H. Lo C. F. (2009). Whispovirus. Curr. Top. Microbiol. Immunol. 328, 197–227. doi: 10.1007/978-3-540-68618-7_6 19216439

[B10] Lightner D. V. Redman R. M. Pantoja C. R. Tang K. F. Noble B. L. Schofield P.. (2012). Historic Emergence, Impact and Current Status of Shrimp Pathogens in the Americas. J. Invertebr. Pathol. 110 (2), 174–183. doi: 10.1016/j.jip.2012.03.006 22434000

[B11] Li J. Ma B. Fang J. Zhi A. Chen E. Xu Y.. (2019). Recombinase Polymerase Amplification (RPA) Combined With Lateral Flow Immunoassay for Rapid Detection of Salmonella in Food. Foods 9 (1). doi: 10.3390/foods9010027 PMC7022641 31887998

[B12] Li W. Wang Q. (2021). Recent Progress in the Research of Exosomes and Dscam Regulated Crab Antiviral Immunity. Dev. Comp. Immunol. 116, 103925. doi: 10.1016/j.dci.2020.103925 33217412

[B13] Mekata T. Sudhakaran R. Kono T. Supamattaya K. Linh N. T. Sakai M.. (2009). Real-Time Quantitative Loop-Mediated Isothermal Amplification as a Simple Method for Detecting White Spot Syndrome Virus. Lett. Appl. Microbiol. 48 (1), 25–32. doi: 10.1111/j.1472-765X.2008.02479.x 19018969

[B14] Mendoza-Cano F. Sánchez-Paz A. (2013). Development and Validation of a Quantitative Real-Time Polymerase Chain Assay for Universal Detection of the White Spot Syndrome Virus in Marine Crustaceans. Virol. J. 10, 186. doi: 10.1186/1743-422x-10-186 23758658 PMC3685563

[B15] Millard R. S. Ellis R. P. Bateman K. S. Bickley L. K. Tyler C. R. van Aerle R.. (2020). How do Abiotic Environmental Conditions Influence Shrimp Susceptibility to Disease? A Critical Analysis Focussed on White Spot Disease. J. Invertebr. Pathol. 107369. doi: 10.1016/j.jip.2020.107369 32272137

[B16] Mustafa M. I. Makhawi A. M. (2021). SHERLOCK and DETECTR: CRISPR-Cas Systems as Potential Rapid Diagnostic Tools for Emerging Infectious Diseases. J. Clin. Microbiol. 59 (3). doi: 10.1128/jcm.00745-20 PMC8106734 33148705

[B17] Park J. Y. Kim K. I. Joh S. J. Kang J. Y. Kwon J. H. Lee H. S.. (2013). Development of a Highly Sensitive Single-Tube Nested PCR Protocol Directed Toward the Sequence of Virion Envelope Proteins for Detection of White Spot Syndrome Virus Infection: Improvement of PCR Methods for Detection of WSSV. Aquaculture 410-411, 225–229. doi: 10.1016/j.aquaculture.2013.06.036

[B18] Piepenburg O. Williams C. H. Stemple D. L. Armes N. A. (2006). DNA Detection Using Recombination Proteins. PloS Biol. 4 (7), e204. doi: 10.1371/journal.pbio.0040204 16756388 PMC1475771

[B19] Schneider L. Blakely H. Tripathi A. (2019). Mathematical Model to Reduce Loop Mediated Isothermal Amplification (LAMP) False-Positive Diagnosis. Electrophoresis 40 (20), 2706–2717. doi: 10.1002/elps.201900167 31206723 PMC7163742

[B20] Sullivan T. J. Dhar A. K. Cruz-Flores R. Bodnar A. G. (2019). Rapid, CRISPR-Based, Field-Deployable Detection Of White Spot Syndrome Virus In Shrimp. Sci. Rep. 9 (1), 19702. doi: 10.1038/s41598-019-56170-y 31873150 PMC6928230

[B21] Takemura K. Satoh J. Boonyakida J. Park S. Chowdhury A. D. Park E. Y. (2020). Electrochemical Detection of White Spot Syndrome Virus With a Silicone Rubber Disposable Electrode Composed of Graphene Quantum Dots and Gold Nanoparticle-Embedded Polyaniline Nanowires. J. NanobiotechnoL. 18 (1), 152. doi: 10.1186/s12951-020-00712-4 PMC7590724 33109213

[B22] Tapay L. M. Lu Y. Gose R. B. Nadala E. C. Jr. Brock J. A. Loh P. C. (1997). Development of an *In Vitro* Quantal Assay in Primary Cell Cultures for a non-Occluded Baculo-Like Virus of Penaeid Shrimp. J. Virol. Methods 64 (1), 37–41. doi: 10.1016/s0166-0934(96)02142-8 9029528

[B23] Trung N. T. Son L. H. P. Hien T. X. Quyen D. T. Bang M. H. Song L. H. (2022). CRISPR-Cas12a Combination to Alleviate the False-Positive in Loop-Mediated Isothermal Amplification-Based Diagnosis of Neisseria Meningitidis. BMC Infect. Dis. 22 (1), 429. doi: 10.1186/s12879-022-07363-w 35508977 PMC9066958

[B24] Tsai Y. L. Wang H. C. Lo C. F. Tang-Nelson K. Lightner D. Ou B. R.. (2014). Validation of a Commercial Insulated Isothermal PCR-Based POCKIT Test for Rapid and Easy Detection of White Spot Syndrome Virus Infection in Litopenaeus Vannamei. PloS One 9 (3), e90545. doi: 10.1371/journal.pone.0090545 24625894 PMC3953118

[B25] van Hulten M. C. Witteveldt J. Peters S. Kloosterboer N. Tarchini R. Fiers M.. (2001). The White Spot Syndrome Virus DNA Genome Sequence. Virology 286 (1), 7–22. doi: 10.1006/viro.2001.1002 11448154

[B26] Waiwijit U. Phokaratkul D. Kampeera J. Lomas T. Wisitsoraat A. Kiatpathomchai W.. (2015). Graphene Oxide Based Fluorescence Resonance Energy Transfer and Loop-Mediated Isothermal Amplification for White Spot Syndrome Virus Detection. J. Biotechnol. 212, 44–49. doi: 10.1016/j.jbiotec.2015.08.003 26277651

[B27] Xia X. Yu Y. Weidmann M. Pan Y. Yan S. Wang Y. (2014). Rapid Detection of Shrimp White Spot Syndrome Virus by Real Time, Isothermal Recombinase Polymerase Amplification Assay. PloS One 9 (8), e104667. doi: 10.1371/journal.pone.0104667 25121957 PMC4133268

[B28] Xiong D. Dai W. Gong J. Li G. Liu N. Wu W.. (2020). Rapid Detection of SARS-CoV-2 With CRISPR-Cas12a. PloS Biol. 18 (12), e3000978. doi: 10.1371/journal.pbio.3000978 33320883 PMC7737895

[B29] Yang H. Ji T. Xiong H. Zhang Y. Wei W. Liu Q. (2022). Transcriptome Profiles of Red Swamp Crayfish Procambarus Clarkii Hematopoietic Tissue in Response to WSSV Challenge. Fish Shellfish Immunol. 122, 146–152. doi: 10.1016/j.fsi.2022.01.041 35124203

[B30] Zhu F. Twan W. H. Tseng L. C. Peng S. H. Hwang J. S. (2019). First Detection of White Spot Syndrome Virus (WSSV) in the Mud Shrimp Austinogebia Edulis in Taiwan. Sci. Rep. 9 (1), 18572. doi: 10.1038/s41598-019-54837-0 31819110 PMC6901514

